# Supplementary material for: The interactome of intact mitochondria by cross-linking mass spectrometry provides evidence for coexisting respiratory supercomplexes
Source: Mol Cell Proteomics. 2017 Dec 8;17(2):216–32. doi: 10.1074/mcp.RA117.000470 (PMC5795388; doi:10.1074/mcp.RA117.000470)
Supplement: Supplemental Data [file supp_RA117.000470_133922_0_supp_23978_fzffwf.zip › spectra_annotation/mito_spectra_annotation/131-1-9-1-9-1.pdf]

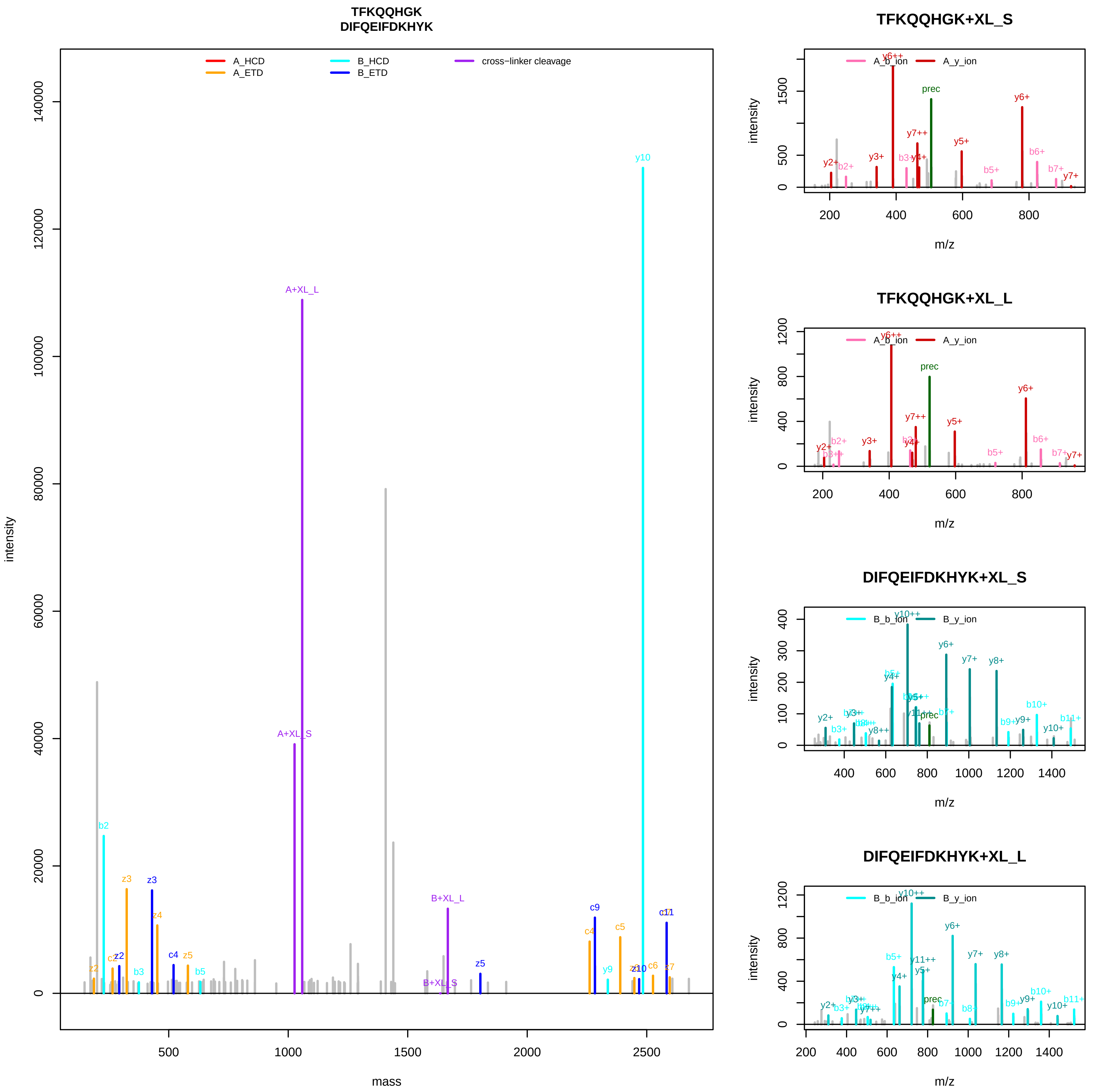
TFKQQHGK
DIFQEIFDKHYK
A_HCD
A_ETD
B_HCD
B_ETD
cross−linker cleavage
intensity
mass
0
20000
40000
60000
80000
100000
120000
140000
500
1000
1500
2000
2500
y10
A+XL_L
A+XL_S
B+XL_L
B+XL_S
b2
z2
z3
z4
z5
c4
b3
b5
z5
c9
c4
c5
y9
z10
c6
c11
z7
TFKQQHGK+XL_S
A_b_ion
A_y_ion
prec
y6++
y6+
y7++
y5+
y4+
y3+
y2+
b2+
b3+
b5+
b6+
b7+
y7+
m/z
TFKQQHGK+XL_L
DIFQEIFDKHYK+XL_S
B_b_ion
B_y_ion
y10++
y6+
y7+
y8+
b10+
b11+
y10+
y9+
b9+
DIFQEIFDKHYK+XL_L
y11++
b5+
y4+
y5+
b8+
